# Deep Learning-Enhanced UV Fluorescence for Automated Detection of Foreign Bodies in Tilapia Fillets

**DOI:** 10.3390/foods15111987

**Published:** 2026-06-03

**Authors:** Huihui Wang, Kangyi Ding, Wenkai Wang, Yuanshan Zhao, Yang Wang, Hao Yuan, Yang Liu, Xiaoyu Xu, Xu Zhang

**Affiliations:** 1 School of Mechanical Engineering & Automation, Dalian Polytechnic University, Dalian 116034, China; whh419@126.com (H.W.); 18623761928@163.com (K.D.); 18317736585@163.com (W.W.); zhaoyuanshan2024@163.com (Y.Z.); 18340826853@163.com (Y.W.); yuanhao@dlpu.edu.cn (H.Y.); yangliu_me@126.com (Y.L.); xuxiaoyu@dlpu.edu.cn (X.X.); 2 State Key Laboratory of Marine Food Processing and Safety Control, Dalian Polytechnic University, Dalian 116034, China; 3 Academy of Food Interdisciplinary Science, Dalian Polytechnic University, Dalian 116034, China

**Keywords:** foreign body detection, ultraviolet fluorescence imaging, U-Net, multi-feature fusion

## Abstract

Tilapia fillets are widely popular worldwide, but endogenous foreign matter (such as scales and bones) remaining during processing poses potential risks to quality control and food safety. Furthermore, these endogenous foreign objects are difficult to detect through manual or traditional visual inspection methods. This study developed a non-destructive rapid detection method for endogenous foreign bodies in tilapia fillets. After acquiring high-quality images of foreign bodies using a UV fluorescence imaging system (360–370 nm), a U-Net deep learning model was first employed to accurately segment the foreign body regions. Subsequently, color features were extracted from various color models (RGB, HSV, L*a*b*, and YCbCr), and texture features were extracted from images enhanced by principal component analysis (PCA). A support vector machine (SVM) classifier optimized using a genetic algorithm was then constructed. Among these, the model integrating color and local binary pattern (LBP) texture features (Color-LBP-GASVM) performed well, achieving an average accuracy of 95.9% and an overall average F1 score of 96.15% on the test set. The results confirm that combining UV-induced fluorescence imaging with an integrated deep learning and machine learning framework holds great potential for the automatic and reliable detection of endogenous foreign bodies in tilapia fillets.

## 1. Introduction

Tilapia is a key source of high-quality, economical whitefish products, and its processing volume has been substantial in recent years, with annual growth. After preprocessing, tilapia is typically cut into fillets or segments for direct sale or used as raw material for further processing [1]. The presence of foreign matter in fillet and segment products is a critical issue affecting food safety and quality [2]. A typical assembly line for whitefish fillets, such as tilapia, includes processes such as deheading, filleting, deboning, trimming, cutting, washing, and quality inspection. Common spray and bubble washing methods can effectively remove most processing residues, such as blood and meat fragments, but they cannot completely eliminate foreign objects like tiny scales and broken bones. In actual production, fish scales and broken bones typically adhere to the surface of the fish meat or are embedded just beneath the surface. Due to their tiny size and translucent color, they are difficult to detect. Currently, the primary detection methods for the aforementioned endogenous foreign bodies include manual inspection, X-ray detection, spectral imaging techniques, and so on [3,4]. Manual inspection relies on visual observation under natural light, and the translucent scales adhering to fish rafts can often only be checked by hand, making it highly susceptible to subjective factors. Furthermore, fish bone fragments may closely resemble the color of fish meat, making accurate visual differentiation quite challenging [5]. Most corporate hygiene protocols require staff to wear rubber gloves, and the chilled state of the raw materials further reduces tactile sensitivity, increasing the risk of missing bone fragments and fish scales. X-ray detection primarily identifies foreign bodies based on density differences and is unaffected by factors such as the color, shape, or position of the fish meat. However, for low-density foreign materials like fish bones and scales, equipment with higher-intensity radiation sources is required, which elevates costs. In some cases, certain fish species with even lower density may remain undetectable [6,7]. In response to this issue, researchers in recent years have proposed utilizing the spectral characteristics of different substances to identify foreign bodies. For example, Song et al. developed a method for detecting fish bones based on Raman hyperspectral imaging technology [8]. Wang et al. used hyperspectral technology to model the spectral differences between fish skin and scales at different wavelengths, thereby achieving a quantitative assessment of the scaling rate of carp [9]. These studies have demonstrated the feasibility of detecting foreign objects by utilizing the differences in light absorption and reflection of fish skin, flesh, and scales within specific wavelength ranges. However, these techniques can only detect fish scales or bones attached to the surface of the fish. Without the introduction of externally excited enhanced imaging technology, it remains impossible to identify foreign objects embedded deep within fish flesh.

Ultraviolet fluorescence imaging (UVFI) with a wavelength range of 320–400 nm (i.e., UVA band or near-ultraviolet light) is an imaging technique based on the molecular fluorescence response generated by materials under UV irradiation [7]. Ultraviolet light in this band possesses strong penetrability and can effectively excite fluorophores in samples. Objects with similar colors exhibit analogous hues within the visible light spectrum (400–760 nm). However, under ultraviolet irradiation, different substances with distinct fluorescence radiation capabilities exhibit different fluorescence reflection intensities. Such intensity differences can improve the contrast of similarly colored targets under ultraviolet excitation and provide favorable detection conditions for foreign bodies hidden in deep fish tissues. For example, Wang et al. combined color and texture features from UV fluorescence images with a CNN model to predict total volatile basic nitrogen (TVB-N) in tilapia subjected to repeated freeze–thaw cycles, thereby demonstrating the feasibility of using fish scales as a carrier of fluorescence information [10]. However, this study focused on predicting quality indicators rather than detecting foreign objects, and it did not address the issue of signal attenuation in deeper layers. In addition, Bao et al. used the UV-press method in conjunction with ISO standard procedures to detect Anisakis simplex in farmed Atlantic cod from Norway, thereby validating the effectiveness of UV fluorescence technology in the detection of foreign bodies in fish [11]. However, this method is destructive, as it alters the original morphology of the sample, and it targets exogenous parasites rather than endogenous foreign bodies in fish. Furthermore, for foreign bodies embedded deep within muscle tissue, the press method struggles to effectively excite and capture fluorescent signals, leaving a risk of missed detection. Although the aforementioned studies did not resolve the issue of non-destructive detection of deep-seated endogenous foreign bodies, they demonstrated the feasibility of UVFI in the identification and quality assessment of foreign bodies in fish. Despite the enhanced contrast offered by UVFI, the intricate biological properties of fish tissues can, in practice, lead to overlapping or ambiguous fluorescence responses between foreign bodies and the surrounding substrate. This similarity in spectral signatures can complicate visual interpretation and traditional image analysis, potentially limiting identification accuracy. Therefore, accurately distinguishing foreign objects from the surrounding matrix has become key to improving recognition performance. The emergence of deep learning has provided a new and effective approach to addressing this challenge. Zhang et al. utilized a U-Net network to achieve accurate identification and segmentation of foreign objects in birds’ nests [12], and Wang et al. employed an improved U-Net model to detect foreign objects on power transmission lines [13]. These studies demonstrate that the U-Net family of models exhibits significant effectiveness in image-based foreign object detection tasks [7]. Their advantage lies in the ability to automatically learn complex and discriminative features from raw image data [14], thereby effectively extracting subtle patterns used to distinguish targets from the background. However, the original U-Net model achieves pixel-level semantic segmentation primarily by learning high-level semantic features within the global context of an image; its classification mechanism relies heavily on deep spatial feature mappings rather than explicit analysis of color and texture features. In the task of identifying foreign objects in tilapia fillets, color and texture differences between various foreign objects serve as critical discriminative information. Both the fish meat itself and foreign objects (especially small fish bones) exhibit fluorescence; although the fluorescence from the fish meat is relatively weaker than that from small fish bones, the small surface area of the fish bones makes them easily obscured by the fluorescent background generated by the fish meat. Although end-to-end models possess strong recognition capabilities, they may still misidentify key features of foreign objects against a broad fluorescent background. In this study, the impact of the fish meat’s fluorescent background on the classification performance of end-to-end models cannot be ignored. [15]. For high-dimensional biological features such as color and texture, previous studies have demonstrated that support vector machines (SVMs) exhibit excellent classification performance. For example, Azarmdel et al. utilized an SVM classifier to achieve high-accuracy automatic classification of four fish species in a fish intelligent processing system [16], while Windarsih et al. employed SVMs to achieve high-precision prediction of adulteration levels in the detection of pork fat adulteration in tuna oil [17]. Therefore, by combining an efficient classifier with these distinctive visual features, it is expected that a robust identification model capable of accurately classifying various types of endogenous foreign bodies can be developed.

**Figure 1:**
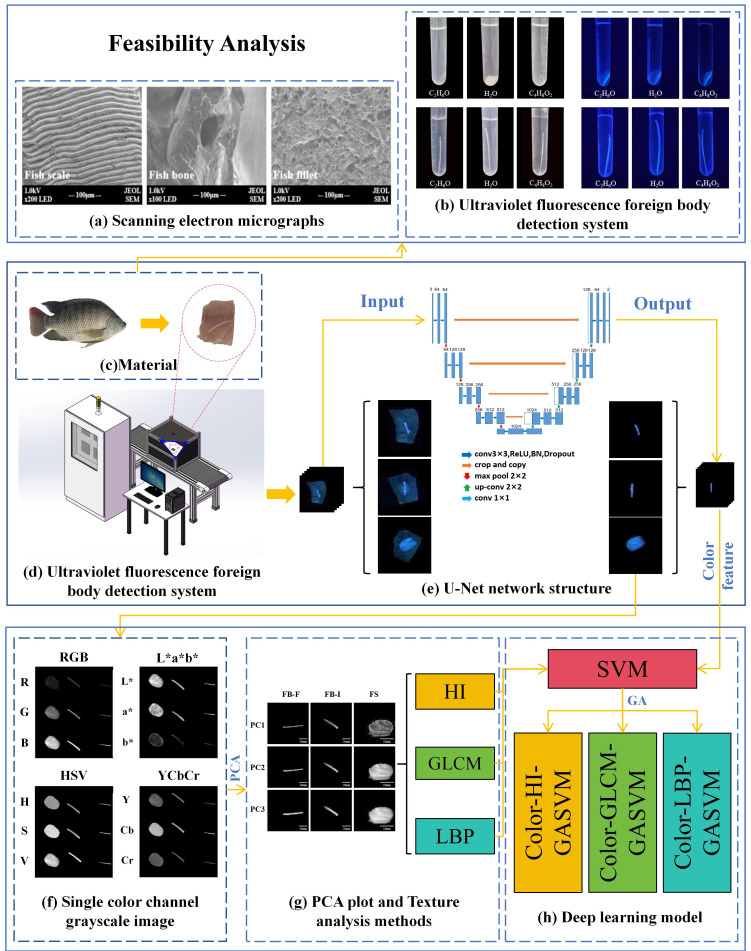
Overall research framework and methodology flowchart.

This study employs ultraviolet excitation technology to enhance the visualization of foreign objects in tilapia fillets. By leveraging the differences in fluorescence intensity between fish meat and foreign objects such as scales and bones under ultraviolet excitation, and by integrating classical machine learning with deep learning methods, a foreign object recognition model for tilapia fillets was developed. The model is designed to enable rapid, non-contact detection of endogenous foreign objects such as scales and bones. The main research content is as follows: Through scanning electron microscopy (SEM) experiments and organic solvent immersion tests, we analyzed the microscopic mechanisms underlying the fluorescence responses of fish scales and bones, thereby verifying the feasibility and reliability of non-destructive foreign object detection in fish fillets based on fluorescence response. We developed a foreign object detection system utilizing ultraviolet fluorescence to capture fluorescence response images of foreign objects and fish fillets. By comparing the performance of basic image processing methods with the U-Net deep learning method, a U-Net was ultimately adopted to achieve high-precision localization of foreign objects in complex backgrounds. Multi-color model space features were fused, and principal component analysis (PCA) was employed for dimensionality reduction. Combining the gray-level co-occurrence matrix (GLCM), local binarized pattern (LBP), and histogram indices (HI), a set of discriminative color and texture features was constructed. By optimizing the classic support vector machine (SVM) machine learning model using genetic algorithms (GAs) and particle swarm optimization (PSO), we achieved rapid and high-precision identification and detection of foreign objects in fish fillets. The overall research framework and methodology flowchart of this study are illustrated in Figure 1.

## 2. Materials and Methods

### 2.1. System Construction and Dataset Establishment

**Figure 2:**
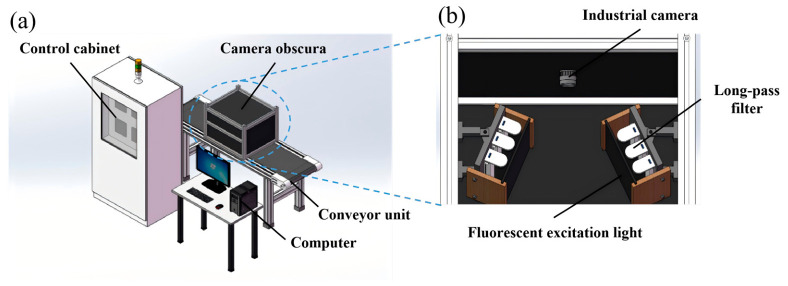
Ultraviolet fluorescence foreign body detection system: (**a**) overhead view of the system; (**b**) internal view of the camera obscura.

In this study, tilapia were purchased in batches from multiple suppliers at the Dalian Xinchangxing Market during different seasons to increase the diversity and complexity of the sample. The purchased tilapia had an average weight of 750 ± 30 g and a body length of 30 ± 3 cm. In response to market demand for frozen fish fillets, we selected fish of a relatively thick size and cut them into fillets approximately 3 mm thick, yielding a total of 585 fillets. To simulate the reality of varying fillet sizes in actual production, this study did not impose any additional restrictions on the length or width of the fillets. The average weight of these fish fillets is 20 g ± 5 g, with a total weight of approximately 9 kg. Among these, 195 fillets had an approximately 1 cm long fish bone inserted horizontally into the mid-thickness layer of the fillet to simulate deeply embedded bone residues, labeled as foreign body FB-I. Another 195 fillets had an approximately 1 cm long fish bone placed on the surface to simulate superficially adherent bone fragments, labeled as foreign body FB-F. The remaining 195 fillets had fish scales placed on the surface to simulate scale adherence, labeled as foreign body FS. To minimize interference with image acquisition caused by production environment noise (such as unstable lighting and background stray light), this study established a foreign object detection system based on UV fluorescence (as shown in Figure 2a). The system comprises a conveyor unit, a computer (Dell), a control cabinet, and a camera obscura. The camera obscura measures 63 × 50 × 44 cm and is constructed with light-impermeable aluminum–plastic panels. As shown in Figure 2b, three strip fluorescent excitation lights (Toshiba Corporation, Tokyo, Japan) with a wavelength range of 360–370 nm were installed on both inner sides of the chamber. The UVA wavelength range (360–370 nm) used in this system has relatively low photon energy and cannot directly break covalent bonds. However, to avoid indirect photooxidation effects that may be triggered by reactive oxygen species (ROS) during prolonged irradiation, we strictly controlled the irradiation time. The actual UV exposure time for each sample was less than 1 s, and the light source was a low-power LED (4 W per lamp). Under such conditions of short irradiation times and low light flux conditions, photochemical oxidation reactions are negligible and do not affect the physicochemical properties of the fish fillet samples, ensuring the non-destructive nature of the test [18]. A long-pass filter (Shanghai Suke Industrial Co., Ltd., Shanghai, China) with a central wavelength of 365 nm was also integrated into the system. The strip fluorescent excitation lights were positioned approximately 20 cm from the sample surface at a 45° angle to the horizontal plane to illuminate the sample. The long-pass filter was mounted parallel to the surface of the light sources. The industrial camera was positioned centrally above the dark box. To effectively control motion blur and ensure consistent image quality, the exposure time was set to 100 μs for capturing top-down images of the sample.

### 2.2. Materials, Instruments, and Reagents

#### 2.2.1. Scanning Electron Microscopy Experiment

Fish meat, scale, and bone samples were frozen in a −80 °C refrigerator for 30 min to solidify internal moisture. Subsequently, they were vacuum freeze-dried for 48 h. Gold coating was applied using the ion sputtering coating method. Finally, cross-sectional images of the fish meat and fish bone samples, as well as surface images of the fish scale samples, were acquired using a scanning electron microscope (JEOL Ltd., Tokyo, Japan) under conditions of −140 °C temperature and 1.0 kV accelerating voltage.

#### 2.2.2. Organic Solvent Immersion and Fluorescence Excitation Tests

Following Qiu Xinjing’s methodology, organic solvent immersion and fluorescence excitation tests were conducted on fish scales and bones [19]. These experiments confirmed that the fluorescence response observed in these materials primarily originates from their intrinsic physicochemical properties (i.e., molecular electronic transitions), rather than external factors such as environmental influences or staining. At room temperature (25 ± 2 °C), wash the fish bone and fish scale samples three times with deionized water (30 s each time). After washing, the excess moisture was gently blotted from the samples with filter paper and then placed into two separate test tubes. Subsequently, 8 mL of absolute ethanol (analytical grade, AR, purity ≥ 99.7%, Sinopharm Chemical Reagent Co., Ltd., Beijing, China) was added to each tube, followed by gentle shaking to ensure thorough contact between the samples (fish scales/bones) and the organic solvent. After standing for 20 min, the samples were placed in a dark environment and irradiated using a 365 nm handheld UV analyzer (Lichen Technology Co., Ltd., Hangzhou, China). Subsequently, the fluorescence response of the fish bones, fish scales, and their respective supernatants was observed under UV irradiation. A visible light control experiment was also conducted; photographs of the control group were taken under indoor ambient light conditions without UV irradiation. The ethanol solvent was replaced with distilled water (prepared in our laboratory) and ethyl acetate (analytical grade, AR, purity ≥ 99.5%, Sinopharm Chemical Reagent Co., Ltd., Beijing, China), respectively, and the above steps were repeated.

### 2.3. U-Net Network Architecture

**Figure 3:**
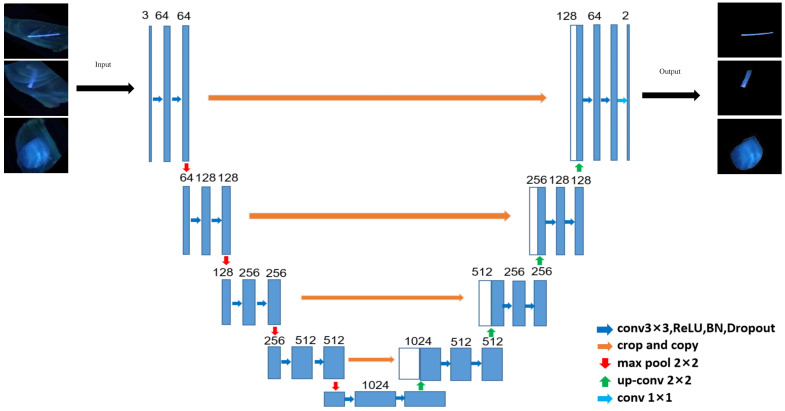
U-Net network structure.

Achieving segmentation of complex background images, such as fish scales, fish bones, and fish fillets, is crucial for implementing foreign body identification. Compared to traditional background segmentation methods such as the thresholding method, the U-Net network can automatically extract features from images. Not only does it demonstrate superior performance when processing complex sample images, but it also effectively mitigates interference caused by changes in the position and orientation of fish fillets due to vibrations or other environmental factors, thereby enhancing the model’s robustness and generalization capabilities in real-world production environments [13,14]. U-Net is a convolutional neural network (CNN) architecture originally designed for biomedical image segmentation [20]. The fundamental structure of the U-Net network consists of three main components: encoding, decoding, and skip connections. Figure 3 shows the architecture of the U-Net network. The left side represents the encoding section, which follows a five-layer framework composed of convolutional layers and pooling layers. The convolutional layers are designed to progressively extract deep semantic information from the fluorescence images of fish fillets. The pooling layers perform downsampling operations to reduce redundant information in the features extracted by the convolutional layers and expand the network’s receptive field relative to the input images. The right side constitutes the decoding section, which exhibits a symmetrical network structure to the left and consists of transposed convolutional layers and convolutional layers. The convolutional layers are employed to extract features from the image and integrate fillet information transmitted from the encoding section via skip connections. The transposed convolutional layers function to progressively restore the image resolution lost during downsampling. Finally, all pixels in the fillet image are classified through a 1 × 1 convolutional layer in the network, thereby achieving segmentation of foreign body regions [21]. Therefore, this study employs the U-Net architecture to perform pixel-level image segmentation. The mask uses a single-channel binary label matching the dimensions of the original image, with the target region assigned a value of 1 and the background assigned a value of 0. The dataset is randomly split into training, validation, and test sets in a 6:2:2 ratio. Training employs binary cross-entropy loss (BCELoss) with the Adam optimizer, a default learning rate of 1 × 10^−3^, a batch size of 4, and a maximum of 350 training epochs, using early stopping at a fixed epoch. For regularization, the network incorporates built-in dropout and batch normalization layers. During training, the loss is recorded every 5 batches, and model weights are saved every 50 batches.

### 2.4. Characterization of Color Features

Color and texture are important intrinsic characteristics of an image. Under the ultraviolet fluorescence excitation system, fish scales and fish bones exhibit varying intensities of blue fluorescence and distinct textural roughness. The surface of fish scales contains ridge lines and grooves, while the surface of fish bones presents ridge-like elevation lines arranged concentrically around the focal point of the scale. In contrast, the surface of fish bones is smooth and exhibits a columnar hollow structure. Based on these discrepancies between fish scales and fish bones, the color and texture features were extracted from the images of foreign bodies, which were segmented by the U-Net algorithm to establish a detection model for foreign bodies in fish fillets.

To comprehensively characterize the color features of foreign body images, four common color models were established: RGB, HSV, CIE L*a*b* (hereafter L*a*b*), and YCbCr. The RGB color model is the most widely used color model in image processing, where R, G, and B represent red, green, and blue, respectively. As a fundamental and highly versatile model, it provides raw pixel-level color information [22]. The HSV color model is designed based on human perception of color, where H (Hue, ranging from 0° to 360°) represents color information, S (Saturation) denotes color purity, and V (Value) indicates brightness. Its property of relatively decoupling color attributes from brightness facilitates the identification of subtle foreign bodies with specific colors [23]. The L*a*b* color model is established based on physiological characteristics. Compared to the RGB color model, it defines a wider range of colors and is independent of external lighting conditions and devices, thereby avoiding issues such as color loss during image processing [24]. In the YCbCr color model, Y represents the luminance component, Cb denotes the blue chrominance component, and Cr signifies the red chrominance component. The luminance component in the YCbCr color model is independent of the two chrominance components, effectively preventing luminance factors from affecting color characteristics and highlighting the chrominance features of foreign bodies against the textured background of fish meat. For the four color models (RGB, HSV, L*a*b*, and YcbCr), targeting the twelve color channels, namely ‘R’, ‘G’, ‘B’, ‘H’, ‘S’, ‘V’, ‘L’, ‘a’, ‘b’, ‘Y’, ‘Cb’, and ‘Cr’, this study considers only the foreign object regions segmented by U-Net as the valid computational domain. The average pixel values for each channel within this region are calculated, and these values are used as the color features for the corresponding channels. The feature calculation formula is as follows:

(1) $$m=\frac{\sum i\sum jf\left(i,j\right)}{N}$$

where m represents the color feature value of the foreign body image, N denotes the total number of pixels in the foreign body image, and f(i, j) represents the single-channel color value at the position (i, j) in the foreign body image. To visually represent color characteristics, the 12 monochrome channel images were subjected to visualization processing. Since redundant information exists across different channels, it may affect model accuracy. Therefore, this study employs principal component analysis (PCA) to perform dimensionality reduction on images from 12 color channels. First, a PCA model is fitted on the training set to learn the global feature space; then, this model is used to perform uniform dimensionality reduction on the training, validation, and test sets, ensuring that the features of all samples reside in the same representational space. Unlike applying PCA to 12-dimensional mean vectors, this study directly applies PCA to the pixel-level matrices of the 12 images. The resulting principal component images fully preserve the spatial structural information of the images. Finally, principal component images that account for 99% of the cumulative variance are selected for subsequent texture feature extraction.

### 2.5. Texture Feature Extraction

For the principal component images obtained in Section 2.5 of the paper, texture features are extracted using the gray-level co-occurrence matrix (GLCM), local binary pattern (LBP), and Histogram Indices (HI). The gray-level co-occurrence matrix was derived by statistically analyzing the co-occurrence frequencies of different grayscale values. For the foreign body images, GLCM texture features—including Correlation (Cor), Contrast (Con), Entropy (Ent), and Energy (ASM)—were extracted along four directions: 0° (horizontal), 45° (main diagonal), 90° (vertical), and 135° (secondary diagonal). A total of 16 features were extracted. The feature calculation formulas are as follows:

(2) $$Con=\sum_{i=1}^{k}\sum_{j=1}^{k}{\left(i-j\right)}^{2}t(i,j)$$

(3) $$ASM=\sum_{i=1}^{k}\sum_{j=1}^{k}{t(i,j)}^{2}$$

(4) $$E_{n}=-\sum_{i=1}^{k}\sum_{j=1}^{k}t(i,j)log\;t(i,j)$$

(5) $$Cor=\sum_{i=1}^{k}\sum_{j=1}^{k}\frac{(i-a_{r})(j-a_{c})t(i,j)}{\sigma_{r}\sigma_{c}}\;\sigma_{r}\neq 0,\sigma_{c}\neq 0$$

where a_r_ and a_c_ represent the mean of the rows and columns of the GLCM, respectively; σ_r_ and σ_c_ denote the standard deviations of the rows and columns of the GLCM, respectively; i and j indicate the grayscale values of paired pixels; t(i,j) is the occurrence count of pixel pair (i,j) observed in the input image [25].

The HI algorithm reflects the statistical characteristics of image texture by analyzing the frequency of occurrence of each gray level in the image. Using the HI method, six primary texture features of the image are extracted: Mean (m) represents the average grayscale brightness of the image; Standard Deviation (σ) reflects the overall contrast and dispersion of grayscale distribution; Uniformity (U) measures the consistency and concentration of grayscale distribution; Entropy (e) describes the randomness and complexity of grayscale distribution; Smoothness (R) characterizes the visual smoothness of the grayscale distribution; and Third Moment (µ) quantifies the skewness of the grayscale distribution. The feature calculation formulas are as follows:

(6) $$m=\sum_{i=0}^{L-1}h_{i}P(h_{i})$$

(7) $$\sigma =\sqrt{\sum_{i=0}^{L-1}{\left(h_{i}-m\right)}^{2}P(h_{i})}$$

(8) $$U=\sum_{i=0}^{L-1}{P(h_{i})}^{2}$$

(9) $$e=-\sum_{i=0}^{L-1}P(h_{i}){log}_{2}P(h_{i})$$

(10) $$R=1-{\left(1+\sigma^{2}\right)}^{-1}$$

(11) $$P(h_{i})=\frac{h_{i}}{N},i=0,1,2,\cdots L-1$$

(12) $$\mu =\sum_{i=0}^{L-1}{\left(h_{i}-m\right)}^{3}P(h_{i})$$

where h_i_ represents the number of pixels with a grayscale value of i; P(h_i_) stands for the normalized occurrence probability of grayscale h_i_ across the whole image; N denotes the total number of pixels in the image block; and L indicates the number of grayscale levels [26].

Local binary pattern (LBP) is a method for describing image texture by comparing the grayscale level of each pixel in the image with those of its surrounding pixels [27]. Specifically, if the grayscale value of the central pixel is higher than that of a neighboring pixel, the neighboring pixel is assigned a binary value of “1”; otherwise, it is assigned “0”. Thus, the eight binary values around each pixel form an 8-bit binary number, which is converted into an integer between 0 and 255, known as the LBP value. This value represents the local texture information of the pixel. However, the original LBP method generates high-dimensional feature vectors containing substantial redundant information, leading to high computational complexity and slow processing speeds. To address this issue, the uniform LBP method proposed by Pietikäinen et al. was adopted to improve and optimize the LBP method [28]. This approach reduces the original 256-dimensional LBP features in a 3 × 3 region to 59-dimensional feature vectors, consisting of p(p − 1) + 2 uniform patterns and one pattern for non-uniform transitions.

### 2.6. Classification Model Establishment

A support vector machine (SVM) is a classical supervised machine learning algorithm. Its core idea is to identify an optimal hyperplane that separates samples of different classes by maximizing the margin between them [29]. SVM demonstrates strong applicability to small datasets and is less prone to overfitting. Leveraging these advantages, it exhibits significant strengths in handling nonlinear, small-sample, and high-dimensional classification tasks [30]. This study selected SVM as the classifier for the classical machine learning-based foreign body detection model in fish fillets [31]. The SVM classification model employed in this research utilizes the radial basis function (RBF) kernel. The critical factors influencing the SVM model’s performance are the error penalty parameter “c” and the kernel parameter “g”, as their selection directly determines the classification accuracy and generalization capability of the SVM [32]. Following the approach used by Wencheng Huang et al. in small-sample classification tasks, this study employs a genetic algorithm (GA) to determine the optimal values of “c” and “g” in the kernel function, using validation set accuracy as the criterion [29]. In particular, the search range for c is [0.1, 100], and the search range for g is [0.001, 3]; the population size of the genetic algorithm is 20, the maximum number of generations is 10, the crossover probability is 0.4, and the mutation probability is 0.1. The algorithm terminates when the maximum number of iterations is reached. Based on the optimized SVM classifier, three foreign object detection models targeting different texture features were developed: Color-GLCM-GASVM, Color-HI-GASVM, and Color-LBP-GASVM.”

A total of 585 original foreign body images were obtained through segmentation using the U-Net network. For each category of foreign object, this study employed stratified random sampling with a fixed random seed of 42, randomly selecting 105 samples for the training set and assigning the remaining 90 samples to the test set. During model training, 20% of the samples were randomly selected from the training set to form a validation set, enabling real-time monitoring of the training progress and dynamic adjustment of model parameters. Given the relatively limited size of the remaining training set and to enhance the model’s robustness to variations in the orientation and position of foreign objects on fish fillets, data augmentation was performed on the training set using a 30-degree horizontal shearing method, effectively doubling the number of training samples. To prevent data leakage and ensure the objectivity of the evaluation, neither the validation set nor the test set underwent data augmentation; instead, the original samples were retained. After augmentation, the training set contained a total of 504 samples (168 per class), while the validation set contained 63 samples (21 per class), and the test set contained 270 original samples (90 per class).

### 2.7. Evaluation Metrics

Intersection over Union (IOU) and segmentation time per image were employed to evaluate the performance of both foundational image processing and the U-Net method. IOU is the ratio of the intersection to the union between the ground truth position of the foreign body region and the model-predicted position. MIOU represents the average IOU across all foreign body images. In Formula (13), TP denotes the number of pixels correctly predicted as foreign bodies, FP indicates the number of pixels incorrectly predicted as foreign bodies, and FN refers to the number of undetected foreign body pixels.

(13) $$IOU=\frac{TP}{TP+FP+FN}$$

(14) $$MIOU=\frac{1}{n}\sum_{i=1}^{n}{IOU}_{i}$$

The confusion matrix is a commonly used performance evaluation tool in classification tasks, designed to compare the predicted categories of a model with the actual categories. It provides a clear visualization of the classifier’s performance for each category [33]. Based on this matrix, several quantitative metrics, including accuracy, precision, and the F1 score, were employed to assess the model. This study employs accuracy, precision, and the comprehensive evaluation metric (F1 score) to assess the model. Among these, accuracy measures the overall correctness of the model’s predictions; precision represents the proportion of samples predicted as a certain category that actually belong to that category; and F1 score is used to comprehensively evaluate the model’s classification performance for individual categories. Higher accuracy and precision values indicate better classification capabilities of the model. The F1 score ranges from 0 to 1, and the closer it is to 1, the better the model’s predictive performance. The calculation formulas are as follows:

(15) $$Accuracy=\frac{\left(P_{xx}+P_{yy}\right)}{\left(P_{xx}+P_{xy}+P_{yy}+P_{yx}\right)}$$

(16) $$Precision=\frac{P_{xx}}{\left(P_{xx}+P_{yx}\right)}$$

(17) $$F1=\frac{{2P_{xx}}^{2}}{\left(2P_{xx}+P_{yx}+P_{xy}\right)}$$

where P_xx_ represents the number of samples of class x predicted as class x, P_yy_ represents the number of samples of class y predicted as class y, P_yx_ represents the number of samples of class y predicted as class x, and P_xy_ represents the number of samples of class x predicted as class y.

## 3. Results and Analysis

### 3.1. Microstructural Analysis of Fish Scales, Bones, and Muscle Tissue

**Figure 4:**
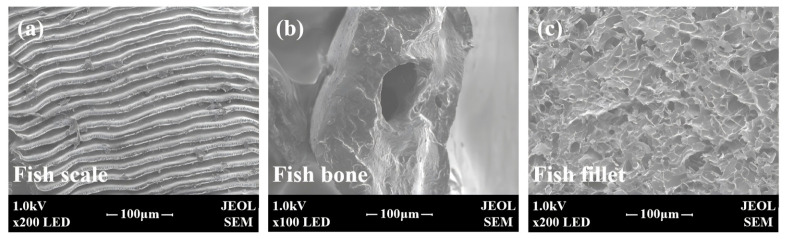
Scanning electron micrographs of different fish body components. (**a**) Morphological microstructure of fish scale; (**b**) Morphological microstructure of fish bone; (**c**) Morphological microstructure of fish fillet.

Figure 4a–c show the scanning electron microscopy (SEM) images of fish scales, fish bones, and fish meat, respectively. As seen in Figure 4a, the scale patterns on the surface of the fish scale form raised ridge-like structures of the bony layer, arranged densely and uniformly with specific angular orientation and distinct directionality. In Figure 4b, the fish bone, formed through the ossification of myoseptal connective tissue, exhibits a hard exterior and hollow interior, primarily composed of carbonates, crude protein, phosphorus, and other substances [34], with a densely packed and compact structure. In contrast, Figure 4c reveals that fish meat consists mainly of muscle fibers with relatively large inter-fiber gaps and a loosely organized structure. The presence or absence of fluorescence in these tissues is governed by two fundamental conditions: the frequency of the incident radiation must be compatible with the molecular structure, and the material must possess a sufficient fluorescence quantum yield after energy absorption at specific wavelengths [35]. This explains why fish bones and fish scales, sharing compositional and structural similarities with their layered inorganic structures, effectively absorb and re-emit ultraviolet light, fulfilling both conditions to produce distinct fluorescent responses. Conversely, the myofibrillar protein structure of fish meat, despite absorbing ultraviolet light, fails to satisfy the second condition, as it does not efficiently re-emit the energy as longer-wavelength light, resulting in a weak fluorescent response.

### 3.2. Organic Solvent Immersion Experiment

**Figure 5:**
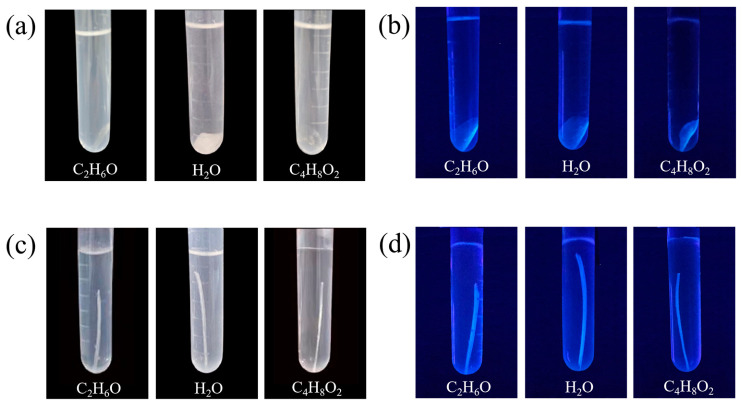
Fluorescence response images: (**a**) Images of fish scales in different solvents under visible light irradiation. (**b**) Images of fish scales in different solvents under ultraviolet light irradiation. (**c**) Images of fish bones in different solvents under visible light irradiation. (**d**) Images of fish bones in different solvents under ultraviolet light irradiation.

Figure 5 shows the fluorescence response diagrams of fish scales and fish bones in different solvents under ultraviolet and visible light. Figure 5a,b depict fish scales and fish bones placed in test tubes containing three different solvents (C_2_H_6_O, H_2_O, and C_4_H_8_O_2_) under visible light irradiation, while Figure 5c,d show the corresponding images under ultraviolet light irradiation. Under natural light, neither fish scales nor fish bones exhibit a fluorescence response. Under ultraviolet light irradiation, only the fish scales and fish bones demonstrate a fluorescence response, while the supernatant in the solution shows no fluorescence. This indicates that the fluorescence is caused by the structural coloration of the fish scales and fish bones and is not influenced by external factors such as feeding conditions or environments, ensuring the stability and reliability of detection in the ultraviolet wavelength range. The differences in fluorescence response between fish flesh, scales, and bones also demonstrate the feasibility of implementing the method described in this paper.

### 3.3. Segmentation Results and Comparative Analysis

**Figure 6:**
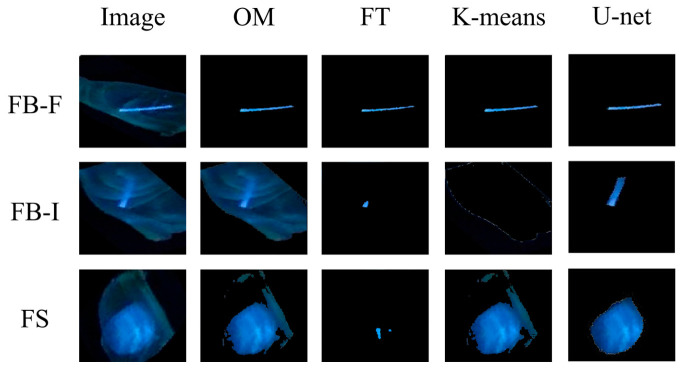
Comparative segmentation results of four image processing techniques applied to fluorescent images of the foreign body FS, FB-F, and FB-I. From left to right: original fluorescent image, segmentation results obtained using the Otsu method (OM), fixed thresholding (FT), K-means, and U-Net.

The segmentation results based on the U-Net network and classical threshold segmentation techniques are shown in Figure 6. The first column displays the collected fluorescent images of fish fillets, while columns 2–5 present the segmentation results obtained by 4 different methods. These involve performing a bitwise AND operation between the mask images generated by each method and the original image to isolate fish scales and bone fragments. Since the foreign body FB-F is located on the surface layer of the fish meat, it exhibits a significant difference in fluorescence response compared to the background fish meat [36]. All four methods can extract the foreign body to some extent, and the IOU data of all tested segmentation algorithms are listed in Table 1. Among them, the fixed threshold method (threshold set to 125) shows obvious discontinuities in the segmentation results, while the Otsu method and K-means method exhibit burrs and pits in the extraction of fishbone edges. The U-Net network achieved a more complete and smooth segmentation with an Intersection over Union (IOU) of 92.6%. For the foreign body FB-I embedded within fish flesh, partial light reflection is absorbed by the flesh, weakening the fluorescence response. Consequently, classical threshold segmentation methods show significantly reduced performance: the Otsu method incorrectly identifies the entire fish fillet as foreign matter, the fixed threshold method detects only sparse bright regions, and K-means fails to identify it entirely with an IOU of 0.0%. In contrast, U-Net still achieves relatively complete segmentation of this foreign body with an IOU of 90.8%. For FS foreign body detection, the transparent nature of fish scales allows their fluorescence images to reveal the underlying texture of the fish meat. Consequently, the Otsu method and K-means both misclassify the fish meat in the edge regions as foreign bodies, while the fixed threshold method only identifies a very small number of bright areas, with an IOU of only 1.8%. In contrast, the U-Net network accurately and completely extracted the FS foreign body, achieving an IOU of 95.3%. This study compared three underlying image-processing techniques. Due to varying fluorescence intensities among foreign bodies, threshold segmentation methods failed to accurately distinguish the three object types. However, the U-Net network significantly improved segmentation performance for all three foreign bodies, demonstrating that its adoption for foreign body segmentation is both reasonable and accurate.

### 3.4. Experimental Results of Color and Texture Feature Characterization

**Figure 7:**
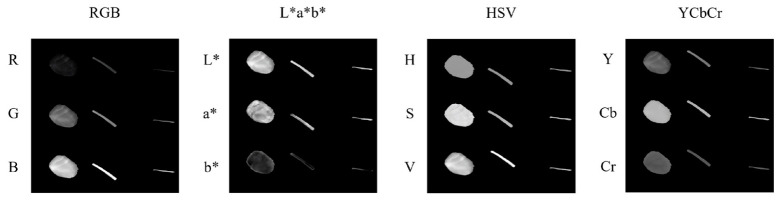
RGB, HSV, L*a*b*, and YCbCr color models with corresponding color channels.

**Figure 8:**
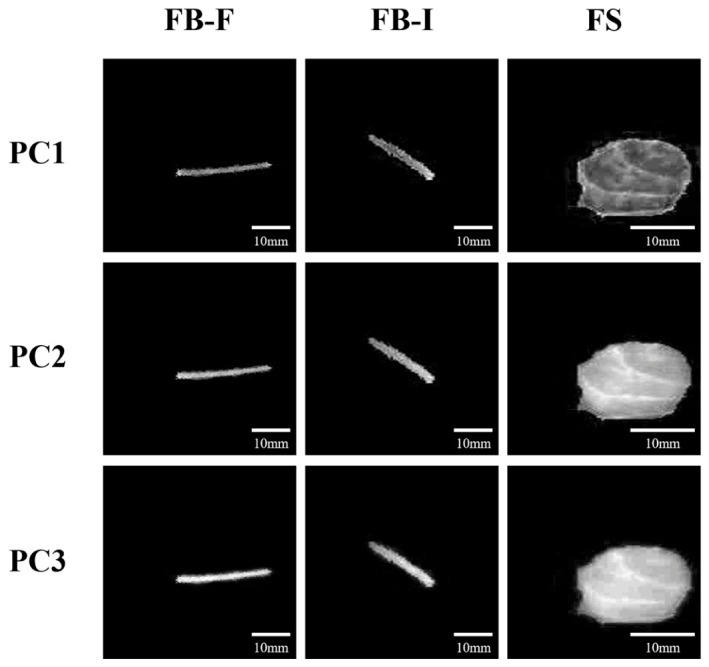
PCA plot of fish scales, fish bones F, and fish bones I.

For the segmented foreign body images mentioned above, a total of 12 single-value features were extracted from individual color channels across the four color models (RGB, L*a*b*, HSV, and YCbCr). As shown in Figure 7, which takes randomly selected foreign body images from the FS, FB-F, and FB-I samples as an example, all components of the four color models are visualized. Figure 7 shows the RGB, L*a*b*, HSV, and YCbCr color models and their respective color channel grayscale images. As can be intuitively observed from the grayscale images of the color channels, the regions and extent of textural prominence in the foreign body area differ across the various channels. This occurs since the individual channels encode distinct discriminative attributes (e.g., color, brightness, hue), causing the perceived textural characteristics of the foreign body to exhibit divergence across them. This difference better highlights the textural features of the foreign body image from multiple perspectives. Additionally, while rich color and texture information exists across different channels, some redundant information negatively impacts model accuracy. Before performing principal component analysis (PCA), the pixel values of the 12 monochrome grayscale images were normalized to the range [0, 1] using the min-max normalization method to eliminate scale differences between channels. PCA was then applied to the normalized grayscale images for dimensionality reduction. Principal component images with a cumulative contribution rate of 99% were selected for further extraction of textural features. Table 2 presents the eigenvalues and contribution rates of the principal components obtained by the PCA method. The cumulative variance contribution rate of the first three principal components (PC1, PC2, and PC3) reached 99.32%, effectively encompassing nearly all the information present in the original data. Figure 8 shows the first three principal component images obtained. It can be observed from the figure that the image clarity of PC1, PC2, and PC3 gradually decreases, indicating that the foremost principal components concentrate most of the information from the original images. Therefore, in this study, PC1, PC2, and PC3 were selected as the primary principal component images for subsequent texture feature extraction.

For the first three principal component images obtained, texture feature extraction was performed using the three methods mentioned in Section 2.6: GLCM, LBP, and HI. Specifically, through the GLCM algorithm, a total of 48 texture features were extracted from each foreign body image (3 principal components × 4 directions × 4 texture features). Through the uniform LBP algorithm, a total of 177 texture features were extracted from each foreign body image (3 principal components × 59 texture features). Through the HI algorithm, a total of 18 texture features were extracted from each foreign body image (3 principal components × 6 texture features).

### 3.5. Optimal Parameters of the Model

**Figure 9:**
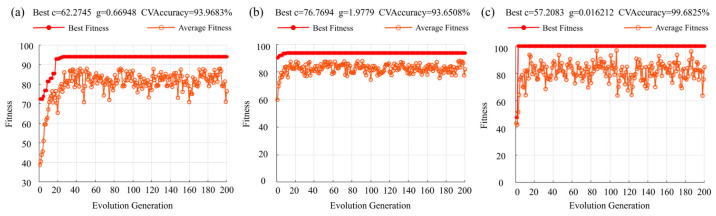
Optimization process of model parameters: (**a**) Color-GLCM-GASVM; (**b**) Color-HI-GASVM; (**c**) Color-LBP-GASVM.

Support vector machine (SVM), a well-established supervised learning algorithm, was selected as the core model for foreign body classification in this study. Its key advantage lies in the ability to project linearly inseparable features from the original space into a higher-dimensional feature space via kernel functions, thereby constructing an optimal separating hyperplane in this transformed space. This characteristic makes it particularly suitable for handling moderate-sized classification tasks with complex feature distributions, such as the one addressed in this research. Therefore, to evaluate the discriminative capability of the extracted texture features and construct high-performance classification models, parameter optimization was performed on the SVM models corresponding to different feature sets. The optimization process employed a genetic algorithm (GA) to optimize the penalty parameter ‘c’ and the kernel parameter ‘g’ of the support vector machine. During optimization, the classification accuracy of the model on the validation set was used as the fitness evaluation metric. As shown in Figure 9a–c, the orange circles and red dots in the figures represent the average fitness and the best fitness values for each iteration, respectively. The best fitness curves of all models converged as the iterations progressed, indicating that the model parameters had reached their optimal solutions. Under these conditions, the parameter combinations corresponding to the highest validation accuracy were selected as the final model parameters. The optimal parameters and their corresponding validation accuracy for each model are presented in Table 3. The Color-LBP-GASVM model achieved the highest validation accuracy of 98.41%, outperforming the Color-GLCM-GASVM and Color-HI-GASVM models (both with validation accuracies below 96%), preliminarily indicating that the Color-LBP-GASVM model possesses superior classification performance.

### 3.6. Classification Results of the Model

In this study, the performance of each model was comprehensively analyzed based on three metrics: validation set accuracy, test set accuracy, and F1 score. As shown in Table 3, the Color-GLCM-GASVM and Color-HI-GASVM models achieved accuracy rates of 95.0% and 93.65%, respectively, on the validation set; however, their accuracy rates on the test set were both below 80.0%, and neither model’s F1 score exceeded 80.0%. These results indicate that while these two models exhibit good fitting performance on the training data, their stability and generalization capabilities are relatively insufficient. Among the three SVM models, the Color-LBP-GASVM model performed the best, achieving accuracy rates of 98.41% and 95.9% on the validation and test sets, respectively, with an F1 score of 96.15%. These results indicate that the Color-LBP-GASVM model demonstrates favorable classification performance in the foreign object recognition task, while also exhibiting reasonable generalization capabilities and model stability. It is worth noting that the model’s accuracy on the validation set is higher than that on the test set, which may suggest a slight degree of overfitting. However, the difference between the two is approximately 2.5 percentage points, which remains within an acceptable range. We believe that the primary cause of this discrepancy is not model overfitting, but rather statistical fluctuations resulting from the random division of small subsets. The validation set consists of 20% of the training set (63 images in total), while the test set comprises 270 independent images. Even if both sets follow the same data distribution, random division under small-sample conditions can still lead to natural fluctuations in accuracy. For example, if the validation set happens to contain more “simple” samples with clear fluorescence images and distinct foreign object edges, while the test set contains more “difficult” samples with complex backgrounds and locally blurred foreign objects, it is reasonable for the validation set to have a relatively higher accuracy than the test set.

**Figure 10:**
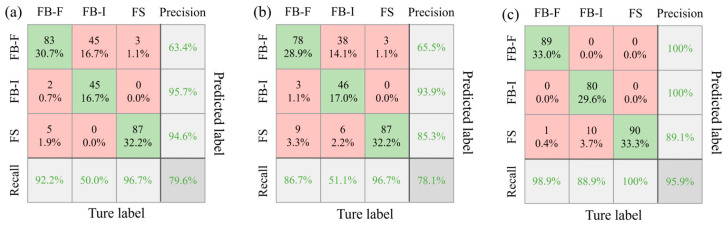
SVM model confusion matrix: (**a**) Color-GLCM-GASVM; (**b**) Color-HI-GASVM; (**c**) Color-LBP-GASVM.

The confusion matrices of the aforementioned models on the test set are shown in Figure 10. As observed in Figure 10a,b, models constructed based on GLCM and HI features exhibit inferior classification performance for both FB-F and FB-I categories. This is primarily because GLCM and HI are global texture statistical features. GLCM extracts the second-order joint probability distribution of an image, characterizing texture by analyzing the spatial co-occurrence relationships between pixel grayscale values across the entire image; in essence, it provides a macroscopic description of the entire image area. HI similarly performs statistical analysis based on global hue, saturation, and intensity distributions. In this study, the foreign object region accounts for a small proportion (approximately 5–15%), while the background is uniformly black (pixel value of 0). Under these conditions, the GLCM co-occurrence matrix is dominated by “0–0” pixel pairs, whose probability is far higher than that of grayscale pairs within the foreign object or at its edges. Consequently, statistical measures such as entropy, correlation, energy, and contrast are primarily determined by the background rather than the foreign object. These global metrics reflect the overall disorder and line complexity of the entire image; as a result, the true texture information of foreign objects is severely diluted or even overwhelmed by a large amount of background data. Similarly, the HI statistics on global color intensity are easily dominated by uniform background regions. Consequently, features extracted based on GLCM and HI contain only weak discriminative information between FB-F and FB-I, resulting in limited classification performance. In contrast, LBP employs a local neighborhood comparison strategy; the LBP code for each pixel depends solely on the gray-level contrast of the 8 pixels within its 3 × 3 neighborhood and is completely independent of the global pixel distribution. For uniform background regions (where all pixel values are 0), the LBP encoding is uniformly quantized to a specific uniform pattern (i.e., one of the LBP uniform patterns). The contribution of this pattern to the LBP histogram is concentrated on a single dimension, rather than generating scattered background noise across multiple statistics as in GLCM. More importantly, through local binarization, LBP is inherently insensitive to illumination and background grayscale, with its encoding results reflecting only relative changes within the local neighborhood [37]. When the proportion of foreign objects is very small, although background regions contain a large number of pixels, they occupy only one or a few feature dimensions in the LBP histogram, whereas the edges of foreign objects, internal microstructures, and local differences between FB-I are encoded into different LBP pattern dimensions. This sparsity effect in feature dimensions enables LBP to effectively suppress global background noise and concentrate limited discriminative information into a few feature channels, thereby significantly enhancing the classifier’s ability to resolve weak signals. Consequently, compared to other classical machine learning models, the Color-LBP-GASVM model not only more accurately identifies FS but also achieves higher classification accuracy for both FB-I and FB-F, with an average model accuracy of 95.9%. In summary, when using color and LBP-based texture features as model inputs, the method demonstrates high classification performance, enabling the model to cover the vast majority of target samples. Among these, the Color-LBP-GASVM model achieved a macro-average accuracy, precision, recall, and F1 score of 96.59%, 96.59%, 96.37%, and 96.15%, respectively. These results indicate that the model performs well in terms of overall classification performance and demonstrates reasonable reliability and potential practicality. Figure 10c shows the confusion matrix for the Color-LBP-GASVM classification model. As shown in the figure, the recall rates for FB-F, FB-I, and FS are 98.9%, 88.9%, and 100%, respectively, with FB-I having the lowest recall rate. In terms of precision, both FB-F and FB-I achieve 100%, while FS has a precision of 89.1%; the model’s overall classification accuracy is 95.9%. The confusion matrix shows that 10 FB-I samples were misclassified as FS, while 1 FB-F sample was misclassified as FS. This may be because FB-I is embedded within the fish meat, with fish tissue adhering to its surface; consequently, the FB-I region segmented by the U-Net includes some fish meat. Additionally, since FS appears colorless and translucent and is attached to the surface of the fish meat, its image exhibits some of the textural features of fish tissue, leading the model to misclassify some FB-I instances as FS during feature analysis. Overall, the Color-LBP-GASVM classification model performs effectively in the classification tasks for the three types of foreign objects.

In summary, the Color-LBP-GASVM classification model achieved the highest accuracy for the three types of foreign bodies (FB-F, FB-I, and FS), with rates of 98.9%, 88.9%, and 100%, respectively. Among these, both FB-F and FS were accurately identified, whereas the classification performance for FB-I was relatively weaker. This may be attributed to the fact that the surface of FB-I is obscured by fish meat, resulting in less distinct color and texture features. The aforementioned results indicate that the approach based on LBP texture features and color characteristics enables effective detection of the three types of foreign bodies and achieves satisfactory classification performance.

### 3.7. Comparative Experiment with End-to-End Models

**Figure 11:**
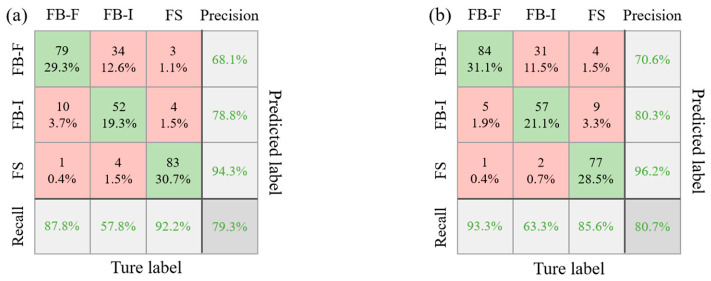
Confusion matrices of end-to-end models: (**a**) ResNet; (**b**) YOLO.

To validate the rationality of the model architecture design, we selected two mainstream end-to-end image classification models (YOLO and ResNet) and conducted direct classification and recognition tests on ultraviolet fluorescence images of three types of foreign objects (FS, FB-F, and FB-I) using the same dataset and training and testing strategies. The experimental results are shown in Figure 11. Compared to the model developed in this study, ResNet and YOLO performed poorly overall in the foreign object identification task for tilapia fillets, with particularly significant shortcomings regarding FB-I-class foreign objects: their recall rates for FB-I were only 57.8% and 63.3%, respectively, with prominent false negatives, making it difficult to reliably capture the features of this type of foreign object. A preliminary analysis suggests that this is primarily due to the inherent fluorescent properties of the fish meat itself. Since foreign objects (especially small fish bones) are relatively small in volume compared to the fish meat background, the end-to-end models may have overemphasized the fish meat background, thereby weakening the key features of the foreign objects and significantly interfering with the classification results.

In contrast, the two-stage architecture proposed in this study (U-Net segmentation, multi-feature fusion, and GASVM classification) first uses a segmentation module to precisely locate foreign object regions and then performs fine-grained classification based on differences in color and texture features. This effectively addresses a shortcoming of end-to-end models, which struggle to capture small, low-contrast features of foreign objects because they learn global features directly from raw images, thereby demonstrating greater validity and applicability in the current application scenarios.

### 3.8. Generalization Evaluation

**Figure 12:**
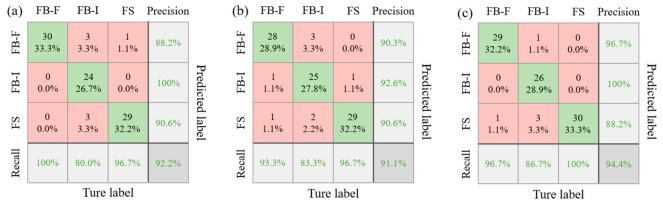
Confusion matrix for different fish species: (**a**) Ctenopharyngodon idella; (**b**) Cyprinus carpio; (**c**) Gadus morhua.

To further evaluate the model’s generalization ability in real-world complex scenarios and prevent it from overfitting to the sample characteristics and background distribution of tilapia, we additionally procured three common whitefish species (Cyprinus carpio, Ctenopharyngodon idella, and Gadus morhua). Using the same sample preparation process as for tilapia, we prepared 90 samples of each fish species, with 30 samples for each of the three foreign object categories, and captured corresponding UV fluorescence images. The image data for these new fish species were directly fed into the pre-trained Color-LBP-GASVM classification model (without any model parameter adjustments or retraining). As shown in Figure 12, the model achieved overall classification accuracies of 92.2%, 91.1%, and 94.4% for Ctenopharyngodon idella, Cyprinus carpio, and Gadus morhua, respectively, all exceeding 90%. However, the recall rate for FB-I class foreign bodies was lower in the three fish species than in tilapia. Preliminary analysis indicates that carp, grass carp, and cod differ from tilapia in terms of muscle protein content and texture. These differences may affect the UV fluorescence response of embedded foreign objects, thereby complicating feature extraction and target recognition and increasing the likelihood of missed detections. Taken together, the results of this cross-species validation demonstrate that the method proposed in this study exhibits reasonable generalizability in detecting foreign bodies within different fish species.

### 3.9. Industrial Applicability and Efficiency Evaluation

To assess the potential value of the detection method described in this paper for industrial applications, this study analyzed the operational efficiency and adaptability of the detection system under dynamic production line conditions. We randomly selected 20 samples from the test set and measured their execution times at each stage of the model. The results showed that the time required to perform precise segmentation of a single foreign object image using the U-Net neural network was consistently less than 365 milliseconds; the time required to extract color and texture features of foreign objects using the feature extraction module was consistently less than 83 milliseconds; and the time required to classify foreign objects using the SVM classification model was consistently less than 0.07 milliseconds. When the processing times of all modules are combined, the entire detection system completes the full identification process for a single foreign object sample in less than 500 ms, generally adapting to the actual production pace of tilapia fillet processing lines; simultaneously, the introduction of a multi-channel parallel detection architecture can further accelerate the process, effectively enhancing the algorithm’s engineering application value. When applying this method to actual production, the “clean/contaminated” classification problem must also be addressed. This can be achieved by directly utilizing the background regions (areas with pixel values of 0) obtained from U-Net segmentation of existing labeled images. By randomly sampling image patches, “foreign-object-free” class samples are constructed and combined with foreign-object region samples to train a simple binary classifier. Furthermore, to address practical operational challenges such as variations in fish fillet thickness and on-site environmental noise, the model can undergo incremental training using actual production data.

## 4. Conclusions

This study employed UV-induced fluorescence technology in conjunction with a deep learning and machine learning integrated network model to preliminarily validate its feasibility in detecting foreign objects in tilapia fillets, thereby offering a new approach to foreign object detection in tilapia processing. Images were collected using a custom-built UVFI system. Leveraging the luminescent characteristics of fish scales and bones under UV fluorescence, a U-Net deep learning segmentation model was constructed to preliminarily achieve the segmentation of foreign body image patches. Color and texture features were extracted from the images of fish scales and bones after U-Net segmentation, and a machine learning prediction model based on a support vector machine (SVM) was established for further classification of foreign bodies. The prediction results indicated that the model constructed based on LBP texture feature extraction and color characteristics exhibited excellent performance (with an F1 score of 95.9%), verifying the effectiveness of this feature combination in foreign body discrimination. The core advantage of this method lies in the fact that UV fluorescence imaging generates high-contrast images that clearly highlight the differences in fluorescence between fish scales, bones, and flesh, with the aim of exploring the potential of fluorescence-based foreign object detection in fish fillets. Although positive results have been achieved under controlled experimental conditions, the current method still falls significantly short of meeting the requirements for practical industrial applications. Future efforts should focus on developing diverse data augmentation strategies to reduce the false negative rate for deeply embedded foreign objects and simulate noise from various complex industrial scenarios, thereby further enhancing the model’s adaptability and reliability in complex production environments. In summary, the method based on UV fluorescence imaging technology and an integrated deep learning–machine learning model shows great promise for foreign object detection in tilapia fillets; however, further research is needed to optimize and validate the approach before it can be applied in actual industrial production.

## Figures and Tables

**Table 1:** Image segmentation results.

Methods	FB-F IOU	FB-I IOU	FS IOU	Mean IOU
OM	52.4%	46.5%	65.0%	54.6%
FT	38.4%	7.9%	1.8%	16.0%
K-means	52.4%	0.0%	65.2%	39.2%
U-Net	92.6%	90.8%	95.3%	92.9%

**Table 2:** PCA principal component results.

PC	Eigenvalue (×10^−4^)	Contribution Rate (%)
PC1	8416.2	84.16
PC2	1246.7	12.47
PC3	268.92	2.69
PC4	65.199	0.65
PC5	2.3472	0.02
PC6	0.2448	0.00
PC7	0.1156	0.00
PC8	0.0539	0.00
PC9	0.0457	0.00
PC10	0.0225	0.00
PC11	0.0000	0.00
PC12	0.0000	0.00

**Table 3:** Parameters and results of the SVM classification model for foreign bodies.

Model	Number of Features	Optimal c	Optimal g	Validation Accuracy (%)	Test Accuracy (%)	F1 Score (%)
Color-GLCM-GASVM	60	62.27	0.67	95.23	79.6	78.8
Color-HI-GASVM	30	76.76	1.97	93.65	91.1	77.1
Color-LBP-GASVM	189	57.21	0.016	98.41	95.9	96.15

## Data Availability

The data presented in this study are available upon request from the corresponding author. The raw data are not publicly available due to pending patent application.
